# Factors Influencing Atypical Clinical Presentations during the 2017 Madagascar Pneumonic Plague Outbreak: A Prospective Cohort Study

**DOI:** 10.4269/ajtmh.19-0576

**Published:** 2020-04-06

**Authors:** Alex P. Salam, Mihaja Raberahona, Prisca Andriantsalama, Liam Read, Faraniaina Andrianarintsiferantsoa, Tiana Razafinambinintsoa, Rado Rakotomalala, Rodrigue N. E. Hasiniatsy, Dominique Razafimandimby, Lyndsey Castle, Anna Funk, Reziky T. Mangahasimbola, Bertrand Renaud, Eric Bertherat, Andrew Lovering, Jean-Michel Heraud, Voahangy Andrianaivoarimanana, Randrianirina Frédérique, Norosoa Razanajatovo, Laurence Baril, Arnaud Fontanet, Minoarisoa Rajerison, Peter Horby, Mamy Randria, Rindra Randremanana

**Affiliations:** 1 University of Oxford, Oxford, United Kingdom;; 2 United Kingdom Public Health Rapid Support Team, London, United Kingdom;; 3 Centre Hospitalier Befelatanana, Antananarivo, Madagascar;; 4 Institut Pasteur de Madagascar, Antananarivo, Madagascar;; 5 North Bristol NHS Trust, Bristol, United Kingdom;; 6 Centre Hospitalier Anti-Pesteux d’Ambohimiandra, Antananarivo, Madagascar;; 7 Centre Hospitalier de Soavinandriana, Antananarivo, Madagascar;; 8 Institut Pasteur Paris, Paris, France;; 9 Université Paris Descartes, Paris, France;; 10 World Health Organization, Geneva, Switzerland;; 11 Conservatoire National des Arts et Métiers, Paris, France

## Abstract

In late 2017, Madagascar experienced a large urban outbreak of pneumonic plague, the largest outbreak to date this century. During the outbreak, there were widespread reports of plague patients presenting with atypical symptoms, such as prolonged duration of illness and upper respiratory tract symptoms. Reported mortality among plague cases was also substantially lower than that reported in the literature (25% versus 50% in treated patients). A prospective multicenter observational study was carried out to investigate potential reasons for these atypical presentations. Few subjects among our cohort had confirmed or probable plague, suggesting that, in part, there was overdiagnosis of plague cases by clinicians. However, 35% subjects reported using an antibiotic with anti-plague activity before hospital admission, whereas 55% had antibiotics with anti-plague activity detected in their serum at admission. Although there may have been overdiagnosis of plague by clinicians during the outbreak, the high frequency of community antibiotic may partly explain the relatively few culture-positive sputum samples during the outbreak. Community antibiotic use may have also altered the clinical presentation of plague patients. These issues make accurate detection of patients and the development of clinical case definitions and triage algorithms in urban pneumonic plague outbreaks difficult.

## INTRODUCTION

On August 27, 2017, a 31-year-old man with pneumonic plague boarded a taxi in the central highlands of Madagascar, heading to the eastern city of Toamasina, via the capital, Antananarivo.^1^ He died on route. A large cluster of infections subsequently occurred among his contacts, with onward transmission in those cities.^1^ The outbreak was eventually declared over on November 27, 2017, after 2,414 suspect cases had been identified (of which 418 were confirmed/probable).^2^ This was the first urban outbreak of pneumonic plague this century.

Plague is caused by the Gram-negative bacteria *Yersinia pestis*. *Yersinia pestis* is endemic in Africa, Asia, and the Americas,^3^ and generally causes two syndromes: bubonic plague and pneumonic plague.^4^ Bubonic plague results from the bite of an infected flea, leading to fever and painful, localized, lymphadenopathy (a bubo).^4^ Pneumonic plague can occur as a result of hematogenous dissemination of bacteria from buboes to the lungs (secondary pneumonic plague), or from human-to-human respiratory transmission via droplets (primary pneumonic plague).^4^ An outbreak of pneumonic plague occurs when an index case with secondary pneumonic plague infects others, resulting in cases of primary pneumonic plague (secondary cases), who in turn themselves infect others (tertiary cases).

Primary pneumonic plague is classically described as presenting with abrupt onset of fever and fulminant progression of respiratory symptoms, characterized by hemoptysis and respiratory distress within 2–3 days.^5^ Untreated mortality is reported to be 100%, whereas treated mortality is reported to be 50%,^5^ with apparently almost all patients succumbing if antibiotics are started 24 hours after symptom onset.^6^ During the Madagascar outbreak, however, there were widespread reports of plague patients presenting with prolonged duration of symptoms (e.g., 1 week and longer), as well as prior histories of viral-like symptoms such as pharyngitis and rhinorrhea. In addition, mortality amongst confirmed patients was lower (25%) than the commonly reported figure of 50%, despite the mean time from symptom onset to presentation being 1.5 days (interquatile range [IQR]: 0–3 days).^2^

There were several potential explanations for these atypical presentations. First, there were widespread reports of people self-medicating with co-trimoxazole during the outbreak, which is readily available over the counter in Madagascar. Co-trimoxazole has activity against *Y. pestis*. However, its use is associated with delayed and incomplete therapeutic responses,^7^ and it is not recommended for the treatment of plague.^8^ Other antibiotics which have activity against *Y. pestis*, such as ciprofloxacin and doxycycline, are also readily available over the counter in Madagascar, as is the case in many countries in which plague is endemic. In addition, primary care and private physicians in Madagascar often administer gentamicin intramuscularly (IM) for patients presenting with infective symptoms. Gentamicin is recommended as a first-line treatment for plague.^8^ However, primary care and private physicians typically administer doses less than 3 mg/kg, which is below the recommended dose for plague (5 mg/kg), and typically only give one or two doses, which is shorter than the recommended duration of 10 days.^8^ We hypothesized therefore that some patients with pneumonic plague were self-medicating and/or being prescribed subtherapeutic short courses of antibiotics in the community, which would delay and alter disease progression, but not actually cure them. This might account for why mortality was actually lower in patients presenting 5 days or more after symptom onset, which is contrary to what is classically described for plague.^6^

A second explanation was that many suspect, and even probable, cases were not due to plague. Antananarivo and Toamasina, which were the focal points for the outbreak, are not endemic areas for plague, and healthcare worker experience of plague in these cities is very limited. Thus, there may have been overdiagnosis by healthcare workers, especially given that the symptoms of pneumonic plague are nonspecific. This might have been exacerbated by the routine use of the F1 antigen rapid diagnostic test (RDT). The RDT is routinely used on bubo aspirates in Madagascar.^9^ However, its use on sputum from suspected pneumonic plague patients has not been formally validated. Poor-quality sputum samples, inexperienced staff, and inapropriate patient selection may have complicated the interpretation of RDT results. Thus, the extent to which it produced false-positive results in patients with non-plague respiratory infections is unknown.

A third possible explanation for atypical presentations was antecedent or coinfection with a respiratory viral pathogen, with a viral prodrome preceding or coexisting with pneumonic plague. Viral respiratory tract infections are known to predispose to concurrent or subsequent bacterial chest infections.^10^

A fourth explanation was that, as for many rare infectious diseases, the mortality and clinical spectrum of pneumonic plague are lower and more diverse than those reported in the literature, with patients presenting with severe “classical symptoms” predominating because of physician awareness and case ascertainment and reporting bias. The descriptions in the literature are almost all retrospective case reports and small case series, with a high risk of bias therefore.^11,12^ Data from an unpublished systematic review suggest that mortality in treated pneumonic plague is approximately 20%, considerably lower than the 50% commonly reported in the literature (Salam, unpublished data).

To identify which, if any, of these factors contributed to the atypical presentation and clinical course of suspected pneumonic plague case in Madagascar in 2017, we conducted a prospective cohort study of patients admitted to pneumonic plague treatment centers. The aim was to describe the clinical, biological, and microbiological characteristics of these patients. The specific objectives of the study were to 1) identify the clinical symptoms and biological markers associated with pneumonic plague; 2) identify the factors (e.g., community antibiotic use and viral coinfection) associated with atypical manifestations of pneumonic plague (e.g., symptom onset > 5 days and symptoms suggestive of viral respiratory tract infection); 3) estimate the case fatality rate among hospitalized pneumonic plague patients, and identify the clinical, therapeutic, and biological factors associated with death; and 4) estimate the sensitivity and specificity of the F1 RDT for diagnosing of pneumonic plague. Although we did not recruit sufficient patients to fulfill these objectives, the results of this study nonetheless provide useful insights.

## METHODS

### Ethics.

This study was approved by the Madagascar National and Oxford University Tropical Research Ethics Committees. All recruited patients or their legal representatives provided informed consent.

### Study design and participants.

Participants were recruited from three hospitals in Antananarivo: Centre Hospitalier Universitaire Joseph Raseta Befelatanana, Centre Hospitalier Anti-Pesteux d’Ambohimiandra, and Centre Hospitalier de Soavinandriana. Patients of all ages admitted to these hospitals with a diagnosis of suspected pneumonic plague were eligible. We did not limit our inclusion criteria to only those patients who met the Ministry of Health (MoH) case definition because the case definition was inconsistently applied across different health centers and by different physicians.

### Data collection.

Each participant was interviewed by a study clinician using a standardized questionnaire. Data collected at admission included demographics, comorbidities, exposure history, symptoms, and antibiotic treatment history. Thereafter, data on respiratory symptoms were collected daily during hospitalization. Data on vital signs, and antibiotic and supportive therapy were retrieved from medical records daily during hospitalization. Data were entered into an electronic case record form on Research Electronic Data Capture by study clinicians.

### Laboratory assessments.

Blood was taken at admission for full blood count, C-reactive protein, blood culture, *Y. pestis* serology, and serum antibiotic levels, and at discharge for *Y. pestis* serology. A nasopharyngeal swab was taken at admission for a multiplex polymerase chain reaction (PCR) assay for respiratory pathogens. Sputum (if productive) was taken at admission for RDT, culture, and *Y. pestis* quantitative PCR (qPCR). For the purposes of estimating the diagnostic value of the RDT, we planned to compare the RDT against culture and serology.

*Yersinia pestis* serology was performed using ELISA, testing for IgM and IgG against F1. Sputum underwent direct culture and amplification in mice followed by biochemical identification by analytical profile index 20E and lysis by bacteriophage. Quantitative PCR targeted the *pla* and *caf-1* genes of *Y. pestis.* Both these targets had to be positive for qPCR to be considered positive. Polymerase chain reaction was performed on nasopharyngeal swabs using the FT1yo respiratory pathogen panel 21, using a cycle threshold cut-off of 37.

Serum was tested for the following antibiotics: co-trimoxazole, doxycycline, ciprofloxacin, and gentamicin. Gentamicin was assayed on an Indiko Plus analyzer using a QMS kit (Thermo Scientific, Waltham, MA). The lower limit of quantitation was 0.3 mg/L; intra-assay precision (as assessed by the coefficient of variation of quality control samples run at the same time) was less than 5%, whereas inter-assay precision was less than 10%. Ciprofloxacin and levofloxacin were assayed by high-performance liquid chromatography (HPLC) on a Spherisorb ODSII 25 cm × 4.6 mm column (Waters, Milford, MA) heated to 50°C. The lower limit of quantification was 0.1 mg/L for both analytes. Recovery was in the range of 90–100%, and intra- and inter-day accuracy and precision were assessed by the use of quality control standards, with limits for accuracy of 15% and coefficient of variability for precision of 10%. Co-trimoxazole (sulfamethoxazole and trimethoprim) was measured by HPLC on a Hypersil 5ODS 100 mm × 4.6 mm column (Thermo Scientific). The lower limit of quantification was 1.0 mg/L for both analytes. Recovery was in the range of 100–110%, and intra- and inter-day accuracy and precision were assessed by the use of quality control standards, with limits for accuracy of 15% and coefficient of variability for precision of 10%. Doxycycline was measured by LCMS-mass spectrometry on an 4000 Q-Trap (AB Sciex, Warrington, England) operating in the positive ion electrospray ionization mode coupled with a Prominence HPLC system (Shimadzu, Kyoto, Japan). Samples were extracted in the ratio of 1:1 with tetracycline-spiked 10% trichloroacetic acid.

### Definitions.

The laboratory definitions of cases were as follows. A confirmed case was defined as: 1) positive RDT performed at Institut Pasteur de Madagascar (IPM) and a positive qPCR, 2) isolation of *Y. pestis* from the culture of a clinical sample, or 3) seroconversion or a 4-fold increase in IgG titers on two different samples. A probable case was defined as: 1) positive RDT performed at IPM, 2) a positive qPCR, or 3) a single serological titer > 0.350. A suspect case was defined as a negative RDT performed at IPM and a negative qPCR and negative cultures and the absence of a serological titre > 0.350.

## RESULTS

From November 3 until December 28, 20 subjects were enrolled into the study. The study recruited low numbers as the outbreak declined rapidly soon after enrollment began. Table 1 details subjects’ symptoms and vital signs at admission and outcomes. Table 2 details *Y. pestis* status, serum antibiotics at admission, antibiotics received during hospitalization, and Ministry of Public Health classification.

**Table 1:** Summary of major clinical findings for each subject at admission

Subject	Gender	Age	Time to presentation	Fever	Cough	Sputum	Bloody sputum	Hemoptysis	Dyspnea	Chest pain	Sore throat	Rhinorrhea	Otalgia	Respiratory rate	Heart rate	Blood pressure (mmHg)	Temperature (°C)	Peripheral oxygen saturation	Level of consciousness
1	Male	30 years	5 days	+	+	−	−	−	−	+	−	−	−	16	89	120/80	40	n/a	A
2	Female	44 years	16 days	−	+	+	−	−	−	−	−	−	−	20	71	100/60	37.2	98	A
3	Female	28 years	18 days	+	+	+	−	−	+	+	−	−	−	32	110	100/70	37.3	94	A
4	Female	39 years	15 days	+	+	+	−	−	−	−	−	−	−	18	87	110/80	37.8	97	A
5	Female	34 years	1 days	+	−	−	−	−	+	−	−	+	−	20	110	90/60	37.1	91	A
6	Male	31 years	24 days	+	+	+	+	−	−	−	−	−	−	22	113	90/60	38.2	96	A
7	Male	11 years	5 days	−	−	−	−	−	−	−	−	−	−	36	80	80/50	37.1	99	A
8	Female	26 years	2 days	+	+	+	+	−	+	+	−	−	−	38	113	110/80	37.4	87	A
9	Male	49 years	0 day	−	+	+	+	−	−	−	−	−	−	22	106	150/100	36.9	96	A
10	Male	66 years	5 days	+	−	−	−	+	+	−	+	−	−	32	140	160/90	38.5	84	Painful stimuli
11	Male	2 years	2 days	+	−	−	−	−	−	−	−	+	−	24	112	n/a	38.5	n/a	A
12	Male	48 years	4 days	+	−	−	−	+	+	+	−	−	−	26	90	100/60	38.3	98	A
13	Female	2 years	0 day	+	−	−	−	−	−	−	−	−	−	36	103	n/a	36	n/a	A
14	Female	45 years	3 days	−	+	+	−	−	−	−	−	−	−	18	90	160/90	36.7	96	A
15	Male	4 months	3 days	+	+	+	−	−	+	−	−	+	−	45	134	n/a	38.4	n/a	A
16	Male	31 years	3 days	+	+	−	+	−	+	+	−	−	−	59	110	110/70	41.5	81	Confused
17	Female	17 years	3 days	+	−	−	−	−	−	+	−	+	−	21	97	100/60	36.8	97	A
18	Male	27 years	19 days	+	+	+	−	−	−	−	−	−	−	32	140	100/60	40.2	94	A
19	Male	55 years	7 days	+	+	+	+	−	+	+	−	−	−	35	110	130/80	37	90	A
20	Male	42 years	2 days	+	+	+	−	−	+	−	−	−	−	22	111	110/70	38.1	91	A

A = alert; n/a: not available; + = positive; − = negative.

**Table 2:** Summary of main laboratory findings and treatment

Subject	Met MoPH case definition	*Yersinia pestis* status	Reported antibiotics	Serum antibiotics	Bld and Spt cultures	White blood cell count (%Neu)	C-reactive protein (mg/L)	PCR Nas	ATB (days)	Outcome (day)	Final classification (MoPH)
1	Yes	NS and SC	Gent	Co-trim and cipro	−	7.73 (86)	134	ADV	Gent (4) and cipro (10)	Alive (D12)	Confirmed
2	No	–	Co-trim	Co-trim	–	9.23 (64)	5	ADV	Strepto (8)	Alive (D8)	Suspected
3	No	–	Co-trim, and gent	Co-trim	–	4.81 (83)	36	ADV	Levoflo (10)	Alive (D9)	Suspected
4	No	–	None	None	–	11,08 (68)	5	ADV	Strepto (8)	Alive (D8)	Suspected
5	No	–	None	None	–	8.22 (67)	41	HRV	Strepto (8)	Alive (D8)	Suspected
6	No	–	Cipro	None	–	13.7 (90)	89	ADV	Strepto (1)*	Alive (D5)	Suspected
7	No	RDT+ and IgG+	Unknown med	Doxy	–	n/a	n/a	ADV/*Sa*	Levoflo (10)†	Alive (D14)	Probable
8	Yes	–	Co-trim	Co-trim	–	11.9 (71)	75	HRV‡/*Sp*	Levoflo (7)	Alive (D8)	Suspected
9	No	–	None	Co-trim	–	7.23 (71)	5	NL63/ADV	Strepto (8)	Alive (D8)	Suspected
10	Yes	RDT+, PCR+, and SC	None	None	*Kp* (Spt)	34.74 (79)	323	HPEV	Gent (3) and levoflo (10)	Alive (D14)	Confirmed
11	No	NS and rest−	None	None	–	n/a	n/a	HRV/*Sp*	Strepto (1) and cipro (2)*	Alive (D2)	Suspected
12	No	–	None	None	–	2.15 (60)	158	Influenza virus A‡/*Sp*	Gent (2), levoflo (2), and ceftri (6)	Alive (D8)	Suspected
13	No	NS and rest−	Gent	None	–	n/a	n/a	ADV‡/*Sp*	None	Alive (D2)	Suspected
14	No	RDT+	Unknown med	None	–	5.39 (62)	16	n/a	Levoflo (5)	Alive (D5)	Probable
15	Yes	–	None	Doxy	*Sa* (Bld)	n/a	n/a	NL63‡/*Sa*	Gent (4) and cipro (4)	Alive (D4)§	Suspected
16	Yes	–	Unknown med	Cipro	–	n/a	n/a	n/a	Gent (1)	Died (D1)	Suspected
17	No	–	Cipro	None	–	4.74 (38)	5	n/a	Strepto (8)	Alive (D8)	Suspected
18	No	–	None	Co-trim	*Sp* (Bld)	11.75 (74)	74	n/a	Strepto (8)	Alive (D8)	Suspected
19	No	–	None	Co-trim and gent	–	25.93 (87)	129	HPEV‡/*Sp*	Strepto (8)	Alive (D8)	Suspected
20	Yes	–	None	Co-trim	–	25.37 (88)	157	ADV	Strepto (8)	Alive (D8)	Suspected

ADV = adenovirus; ATB = antibiotics received during hospital admission (with duration in days); Bld = blood; Cipro = ciprofloxacin; Co-trim = co-trimoxazole; Doxy = serum antibiotics; Gent = gentamicin; HPEV = paraechovirus; HRV = rhinovirus; MoPH = Ministry of Public Health; n/a: not available; NL63 = coronavirus NL63; NS = no sputum; PCR Nas = polymerase chain reaction on nasopharyngeal swab; RDT = rapid diagnostic test; Spt = sputum; Unknown med = unknown medication; %Neu = percentage of neutrophils.

* Antibiotics discontinued.

† Antibiotic started 4 days after inclusion.

‡ Multiple viruses detected on PCR, only virus with the lowest cycle threshold is shown.

§ Self-discharge against medical advice (ciprofloxacin was continued after discharge).

The mean age of subjects was 31.4 (SD 18.1) years, and 12 subjects were male (60%). No subjects reported exposure to known or suspected pneumonic plague cases. The median time from symptom onset to hospital admission was 3.5 days (IQR: 2–13 days), and the median duration of hospital stay was 8 days (IQR: 5.5–8 days).

Nine subjects were treated with streptomycin alone (45%), three with levofloxacin alone (15%), two with gentamicin and levofloxacin (10%), two with gentamicin and ciprofloxacin (10%), one with streptomycin and levofloxacin (5%), one with streptomycin and ciprofloxacin (5%), one with gentamicin alone (5%), and one did not receive antibiotics (5%). Among the two confirmed cases, one received gentamicin for 3 days + levofloxacin for 10 days, and one received gentamicin for 4 days + ciprofloxacin IV for 10 days. One probable case was treated as an outpatient with levofloxacin for 10 days, and the other probable case did not receive any treatment for plague. One suspect patient died.

Seventeen subjects (85%) sought medical care for their illness before admission. Of these, 11 (65%) visited a private doctor, three (17.5%) self-medicated, and three (17.5%) visited a different hospital. Nine (45%) subjects reported antibiotic use before admission, of which seven reported using an antibiotic with activity against *Y. pestis*: two ciprofloxacin, two gentamicin, two co-trimoxazole, and one gentamicin and co-trimoxazole. An additional three subjects took medication before admission, but did not know whether these were antibiotics or not. Eleven (55%) subjects had anti-plague antibiotics detected in their serum at admission: six co-trimoxazole, two doxycycline, one ciprofloxacin, one co-trimoxazole and ciprofloxacin, and one gentamicin and co-trimoxazole. Table 1 details reported antibiotic use before admission and antibiotics detected in serum on admission for each subject. Overall, 14 subjects (70%) either reported community antibiotic use before admission or had antibiotics detected in their serum at admission.

Seventeen patients provided sputum samples. None grew *Y. pestis*, although one sample from the two plague-confirmed patient sputum samples grew *Klebsiella pneumoniae*. All patients had blood cultures and serology performed. One blood culture grew *Streptococcus pneumoniae* and another *Staphylococcus aureus*, both from suspect patients. Two subjects had confirmed *Y. pestis* infection: one had a positive RDT, positive qPCR, and a seroconversion in anti-F1 IgG, and the other had a seroconversion in anti-F1 IgG alone (this subject did not produce sputum). Two subjects had probable *Y. pestis* infection, one having a positive RDT alone and the other having a positive RDT and an anti-F1 IgG of 0.53 at admission. Nineteen patients had a nasopharyngeal swab performed. Sixteen (84%) had atleast one virus detected by PCR, and nine (47%) had either *S. aureus* or *S. pneumoniae* detected by PCR. Both of the confirmed and one of the probable patients had adenovirus detected in their nasopharyngeal swabs.

## DISCUSSION

Although the study recruited a low number of patients because of the outbreak declining rapidly after enrollment began, the results still provide useful insights. Although only four patients were either confirmed or probable cases, the microbiological diagnosis of pneumonic plague is complicated by the fact that it often relies on sputum. Among the two confirmed cases of pneumonic plague (subjects 1 and 10), subject 1 was likely to have an “atypical” presentation of pneumonic plague. This patient’s only symptom at presentation was a nonproductive cough. This therefore did not allow for sputum sample collection and testing. This case was confirmed by seroconversion. This patient’s prior use of antibiotics, ciprofloxacin and co-trimoxazole detected in serum and the reported use of gentamicin before the hospital admission, probably contributed to the paucity of symptoms at presentation. The two probable cases (subjects 7 and 14) remain poorly understood from a clinical point of view. Subject 7 did not present any clinical signs suggestive of pneumonic plague. However, the RDT and a single serology were positive. Similarly, subject 14 had only cough and sputum without fever or signs of respiratory distress. For this case, the RDT was positive, and the serology was negative. This patient had a favorable outcome without any plague treatment. These two cases raise significant questions about the value of the RDT and serology as a diagnostic tool for pneumonic plague, and the possibility of false-positive results.

Poor-quality samples, delays in sample transportation, the non-sterile nature of sputum, and the slow growth of *Y. pestis* in culture render sputum culture an insensitive method.^13^ The sensitivity of PCR in the detection of *Y. pestis* in sputum is also unknown, and it is recognized that the use of the plasminogen activator protein gene as a PCR target for the detection of *Y. pestis* can lead to specificity issues.^14^ The sensitivity (and specificity) of the RDT is also unclear, as are its negative and positive predictive value when used by healthcare workers with limited experience and training. Although we performed serology at admission and discharge on subjects, the time interval between these two samples was often shorter than that recommended (21 days). We were unable to perform serology after discharge because of logistical reasons. Forty percent of subjects in our study presented with bloody sputum. Although this is reported as a classical sign of plague pneumonia,^5^ it is not specific. The clinical presentation of patients and current diagnostic methods do not, therefore, allow even experienced healthcare workers to confidently rule in or rule out cases. Many patients presenting at hospital triage centers may have also exaggerated their symptoms because of fear and panic of possibly having pneumonic plague, and the desire to be treated. In settings such as urban areas, in which healthcare facilities receive large volumes of patients with significant respiratory symptoms, this is likely to lead to high numbers of non-cases being suspected of having pneumonic plague. During the outbreak, this resulted in the healthcare system being overwhelmed with clinical suspect cases. It is difficult to estimate the true rate of infection among these cases, although many probably did not have plague. In general, all suspect patients were treated with a 10-day antibiotic course, irrespective of the results of microbiological investigations. This usually consisted of multiple injections of streptomycin IM per day (the national treatment protocol). Thus, a substantial proportion of patients who probably had bacterial or viral respiratory tract infections, rather than plague, received prolonged courses of a drug which is ototoxic, nephrotoxic, and teratogenic, and which can result in muscle fibrosis when delivered frequently by IM injection.^15^ In fact, most subjects in our study had one or more viruses detected on PCR of nasopharyngeal swabs, and 40% of patients described antecedent symptoms suggestive of a viral respiratory tract infection. Of note, three of four of the confirmed/probable subjects had virus detected on PCR. Whether this predisposed them to infection with *Y. pestis* is unclear. However, viral respiratory tract infections are known to predispose to bacterial chest infections.^10^

Importantly, 55% of subjects in our study had antibiotics detected in their serum at admission, and a further 15% reported taking antibiotics before admission. Most of these subjects procured antibiotics through private doctors or from pharmacies and other shops. Of those who had antibiotics detected in their serum, 55% had only co-trimoxazole detected, and 18% had only doxycycline detected. Treatment of plague with co-trimoxazole is associated with delayed and incomplete therapeutic responses.^7^ Although a small randomized controlled trial suggested that doxycycline resulted in similar cure rates compared with gentamicin for bubonic plague,^16^ many physicians do not use it for pneumonic plague because it is bacteriostatic. Although our study sample size is small and only includes four probable/confirmed plague cases, it is reasonable to assume that antibiotic use would have been just as frequent among the larger outbreak population, as well as specifically those patients with plague. A substantial proportion of patients may, therefore, have taken antibiotics that while having activity against *Y. pestis* are known or suspected to be suboptimal for the treatment of pneumonic plague. The effect that community antibiotic use had on the progression and presentation of disease in patients with *Y. pestis* infection is uncertain. However, it is not unreasonable to assume that the use of co-trimoxazole, and possibly doxycycline, would have altered the timing and manner in which patients presented, further complicating the design of clinical triage algorithms and identification of patients. The effect that prehospital antibiotic use had on *Y. pestis* transmission, by prolonging the duration for which individuals may have been symptomatic in the community, is also unclear. The high frequency of prehospital antibiotic use may, however, partly explain why so few sputum samples grew *Y. pestis* during the outbreak, notwithstanding the diagnostic issues associated with sputum described earlier. During the outbreak, only eight sputum samples grew *Y. pestis*. Although this small number of culture-positive samples may also be as a result of overdiagnosis by clinicians, *Y. pestis* growth is easily impaired by antibiotics to which it is sensitive. In situations in which community antibiotic use is high therefore, PCR and the RDT may be better diagnostic tools than culture. However, this remains to be determined, and the predictive value of these diagnostic tools still needs to be formally assessed.

The high frequency of prehospital antibiotic use that we observed is unlikely to be particular to Madagascar. Antibiotics are widely available over the counter in most countries in which plague is endemic.^17^ Co-trimoxazole, doxycycline, and ciprofloxacin are all readily available in Africa and Asia.^17^ In one study, antibiotics were detected in the urine of 64% of patients at attendance to hospital, with ciprofloxacin and trimethoprim accounting for most of those detected.^18^ As in Madagascar, nonprescription antibiotic courses in most countries are also often shorter and lower in dose than those indicated for specific illnesses,^17^ increasing the risk of incomplete therapeutic responses and the emergence of antibiotic resistance, although none of the cultured strains during the outbreak showed resistance.

Managing an outbreak of pneumonic plague in an urban setting presented numerous challenges and required a complex and large response from the MoH, WHO, and partner organizations. Specifically, the accurate identification and diagnosis of cases proved difficult in an urban setting in which epidemiological context was not discriminative, physicians had no prior experience of pneumonic plague, and antibiotics with activity against *Y. pestis* were readily available in the community, all of which were further complicated by the lack of a validated, sensitive, and specific diagnostic test for pneumonic plague. Similar issues occurred during the outbreak of pneumonic plague in Surat city in India in 1994, in which fear and panic were also widespread.^19^ These issues will invariably repeat themselves in future outbreaks and need to be addressed beforehand. First, more data are needed on the sensitivity and specificity of qPCR and of the RDT when used on patient sputum samples. Although there is no gold standard diagnostic test for pneumonic plague, comparison against serology could prove useful. Data from bubonic plague patients show that most patients develop anti-F1 IgG in response to infection, even when the time interval between samples is as little as 6 days.^20^ Although the data from pneumonic plague patients are much more limited, they nonetheless suggest that a significant number of patients seroconvert.^21^ However, as for other infections, there may be a negative correlation between the extent of infection before therapy and the development of antibodies,^22^ and the widespread prehospital use of antibiotics could limit the applicability of serology. Notwithstanding this, serology should be performed as standard on all patients at admission and discharge, and, where possible, again as an outpatient 21 days after admission. This would not only potentially support the validation of new and existing diagnostic assays but also help better estimate the scale of any outbreak. Second, community engagement needs to occur from the outset of any outbreak and should include targeted messages surrounding the risks of nonprescription antibiotic use (e.g., incomplete treatment, ongoing transmission to the community, and drug resistance). Third, although nonprescription antibiotic use is a long-term problem that requires robust governance structures, monitoring of pharmacies should be instituted during outbreaks as an emergency measure. Fourth, education of healthcare workers, in particular private doctors and general practitioners, should occur from the outset and include education on appropriate antibiotic use and referral processes. Fifth, there needs to be greater emphasis on the clinical and microbiological diagnoses and management of non-plague cases, especially in urban outbreak, and treatment should include antibiotics that cover common causes of community pneumonia as well. The pursuit of differential diagnoses is often neglected in outbreaks, which undoubtedly has negative consequences on the clinical management and psychological health of patients, as well as on the counseling of contacts. Sixth, modeling would be useful to determine whether real-time genetic sequencing would help identify transmission chains^23^ if there were high numbers of culture-positive cases during an outbreak. Seventh, consideration should be given to developing clinical triage algorithms that incorporate information on prehospital antibiotic usage and other factors that may result in atypical presentations. Developing tools, procedures, and protocols that focus on these issues now will enable us to be better prepared and equipped to deal with the next urban outbreak of pneumonic plague.
